# Supplementary material for: Coupling dynamic response of saturated soil with anisotropic thermal conductivity under fractional order thermoelastic theory
Source: PLoS One. 2024 Apr 17;19(4):e0297651. doi: 10.1371/journal.pone.0297651 (PMC11023567; doi:10.1371/journal.pone.0297651)
Supplement: Attachment — Submitted filename: review comments.docx [file pone.0297651.s002.docx]

Title: " Coupling Dynamic Response of Saturated Soil with Anisotropic Thermal Conductivity under Fractional Order Thermoelastic Theory "

Abstract:

This paper presents a significant revision and expansion of the study titled " Coupling Dynamic Response of Saturated Soil with Anisotropic Thermal Conductivity under Fractional Order Thermoelastic Theory." This article addresses A two-dimensional (2D) thermo-hydro-mechanical dynamic (THMD) coupling analysis in the presence of a half-space medium is investigated by using Ezzat’s fractional order generalized theory of thermoelastic. The influence of the anisotropy of thermal conduction coefficient, fractional derivatives, and frequency on the THMD response of anisotropy, fully saturated, and poroelastic subgrade is then analyzed with a time harmonic load including mechanical load and thermal source subjected to the surface by using normal mode analysis (NMA). The general relationships among the dimensionless physical variables including vertical displacement, excess pore water pressure, vertical stress, and temperature distribution are graphically illustrated. NMA method does not need integration and inverse transformation, improves the decoupling speed, and eliminates the limitation of numerical inverse transformation. The calculated results can guide the geotechnical engineering construction according to different values of load frequency, fractional order coefficient, anisotropy of thermal conduction coefficient which improves the stability of subgrade, and enriches the theoretical research in the field of thermo-hydro-mechanical coupling.

I suggest the authors to consider the following encouraging points to further improve the paper presentation and content;

1. **Introduction:**

Revisit the introduction section to provide a more comprehensive background on Fractional Order Thermoelastic Theory application.

2. **Methodology:**

Expand the methodology section to include a detailed description of the numerical approach, numerical methods, and computational tools used in the study.

3. **Model Validation:**

Include a robust validation section that demonstrates the accuracy and reliability of the numerical model. Compare computational results with relevant experimental or clinical data to establish the model's credibility.

4. **Discussion:**

Expand the discussion section to provide more nuanced interpretations of the results. Address the advantages and limitations of Normal mode analysis. Explore potential clinical implications and future research directions.

5. **Conclusion:**

Summarize the major findings and the broader implications of the enhanced study. Highlight the significance of the refined numerical approach.

6. **Language and Clarity:**

Review the paper for language clarity, grammar, and overall readability. Ensure that the flow of the paper is logical and coherent, with smooth transitions between sections.
